# Lack of Association Between Transforming Growth Factor Beta 1 -509C/T and +915G/C Polymorphisms and Chronic Hepatitis B in Iranian Patients

**DOI:** 10.5812/hepatmon.13100

**Published:** 2014-04-07

**Authors:** Armin Hosseini Razavi, Pedram Azimzadeh, Seyed Reza Mohebbi, Seyed Masoud Hosseini, Sara Romani, Mahsa Khanyaghma, Yasin Hatami, Afsaneh Sharifian, Mohammad Reza Zali

**Affiliations:** 1 Gastroenterology and Liver Diseases Research Center, Shahid Beheshti University of Medical Sciences, Tehran, IR Iran; 2 Department of Microbiology, Faculty of Biological Sciences, Shahid Beheshti University, Tehran, IR Iran; 3 Basic and Molecular Epidemiology of Gastrointestinal Disorders Research Center, Shahid Beheshti University of Medical Sciences, Tehran, IR Iran

**Keywords:** Transforming Growth Factor beta 1, Hepatitis B, Chronic, Polymorphism, Genetic

## Abstract

**Background:**

Chronic hepatitis B is one of the world's major health concern. The etiological agent of this infection is hepatitis B virus (HBV), which can evade the immune system response. Transforming growth factor beta 1 (TGF-β1) can act against HBV by suppressing the viral replication. The TGF-β1 also plays an important role in preventing liver damage in chronically HBV infected patients.

**Objectives:**

In this study, the association of TGF-β1 +915G/C and -509C/T gene polymorphisms with chronic hepatitis B was evaluated in Iranian patients.

**Materials and Methods:**

A population-based case–control study was conducted in Taleghani Hospital, Tehran. A number of 220 patients with chronic hepatitis B and the same number of healthy control subjects were designated the case and the control groups. The PCR-Restriction Fragment Length Polymorphism Method (PCR-RFLP) method was used for genotyping both polymorphisms. Ten percent of the control samples were sequenced to confirm the results.

**Results:**

No statically significant differences in genotype distribution and allele frequency were observed for both polymorphisms between healthy controls and patients with chronic hepatitis B.

**Conclusions:**

There was no association between TGF-β1 -509C/T and +915G/C polymorphisms with chronic hepatitis B and it seems that these changes don not play a significant role in increasing the risk of chronic infection in Iranian patients.

## 1. Background

Hepatitis B virus (HBV) is a DNA virus with eight genotypes, of which only genotype D is circulating in Iran (1, 2). Three antigens, including hepatitis B surface antigen (HBsAg), hepatitis B core antigen (HBcAg) and hepatitis B e-antigen (HBeAg) are important in the clinical diagnosis (3-5). The HBsAg can be detected in patients with chronic hepatitis B for more than 6 months (6). In chronic hepatitis B, HBV has escaped from the innate, and also the adaptive immune system, by various mechanisms and, consequently, the immune responses could not successfully control and clear the viral infection (7). Chronic HBV infection may lead to more severe complications, including liver cirrhosis and hepatocellular carcinoma (HCC), which represent life threatening conditions (8).

Transforming growth factor beta 1 (TGF-β1), as the most abundant TGF-β isoform, is a polytropic cytokine which can regulate the immune system and cellular functions. On the other hand, HBV replication can be suppressed directly by TGF-β1. Recently, it has been found that TGF-β1 could reduce the hepatocyte nuclear factor-4-alpha (HNF4A) and this process scales down HBV replication. Also, TGF-β1 has a role in restraining overactive immune responses against HBV infected hepatocytes, therefore preventing liver damage. In these circumstances, TGF-β1 may possibly play a pivotal role in HBV infection (9-11).

Human TGF-β1 gene is located on chromosome 19q13.1-13.3 and has several functional polymorphisms (12, 13). One of TGF-β1 promoter polymorphisms is -509C/T (rs1800469), which has an impact on TGF-β1 expression. In addition, TGF-β1 +915G/C (rs1800471) polymorphism is located at codon 25 of the TGF-β1 protein and the substation of thymidine with cytosine in this location leads to a non-synonymous amino acid change (arginine to proline). Recent studies have revealed that -509C/T and +915T/C polymorphisms are associated with serum TGF-β1 levels (13-17). Since these two TGF-β1 polymorphisms could possibly play a role in an individual's susceptibility to chronic infection and disease progression, their association with chronic hepatitis B and C was investigated in different populations (18-22). However, there is no report on TGF-β1 polymorphisms association with HBV chronic infection in Iranian patients.

## 2. Objectives

In the present survey, the association of TGF-β1 -509C/T and +915T/C polymorphisms with chronic hepatitis B has been evaluated in Iranian patients.

## 3. Patients and Methods

### 3.1. Population

The study was designed based on a case-control methodology. Both the patients and controls groups consisted of 220 individuals. Case and controls sample size was calculated based on the results of previous studies (20) and the formula is presented below:

n = [Z1-α/2 √P0 (1-P0) + Z1-β √Pa (1-Pa)] 2/(Pa-P0)2 as n = 143, Pa = 0.68 and P0 = 0.88, β = 1.2, α = 5% and Power = 80%.

Selection criteria for patient group were Anti-HBc antibody (Anti-HBcAb) and HBsAg positivity for more than 6 months. Most individuals had histories of multiple hospital admissions. Anti-HBcAb positive subjects, after first admission, were investigated for the presence of HBsAg. Both PCR and HBV-DNA viral load tests were conducted on HBsAg-positives cases. The control group consisted of Anti-HBcAb and HBsAg negative individuals, with results confirmed by enzyme-linked immunosorbent assay (ELISA).

### 3.2. DNA Extraction and Genotyping

A 4 mL blood sample was obtained from each person. The DNA isolation from blood samples was performed by the phenol-chloroform extraction method. Genotyping was done by PCR-Restriction fragment length polymorphism (PCR-RFLP) in PCR Thermal Cyclers (Eppendorf AG, Hamburg, Germany). The total volume for a single PCR cycle of either polymorphism was 25 µL. Each single reaction used 100 ng DNA, 2.5 µL buffer, 1.5 µM MgCl_2_, 25 µM of each dNTP and 1.25 U Taq polymerase (Eppendorf AG, Hamburg, Germany). Afterwards, 0.5 µM and 0.7 µM of each primer (Table 1) were added to the -509 and +915 PCR reactions, respectively. The PCR cycle of +915 was performed by a stage of pre-denaturation at 95°C for 5 minutes, then 35 cycles of 95°C for 30 seconds, 58.4°C for 30 seconds, and 72°C for 30 seconds, followed by a ﬁnal extension at 72°C for 10 minutes. The difference between the -509 and + 915 PCR program was the annealing temperature (62.5°C for -509 primers). The resulting PCR products were digested by two enzymes (Eco81I and BglI) in the RFLP reaction at 37°C. Detection of +915G/C and -509C/T polymorphisms was done by BglI and Eco81I (Fermentas, Vilnius, Lithuania), respectively. The RFLP fragments (Table 2) were detected on 3% w/v agarose gel (Hoffmann–La Roche AG, Basel, Switzerland) stained with ethidium bromide. Ten percent of samples were sequenced by ABI genetic analyzer 3130xl to confirm the results of genotyping.

**Table 1.** Primers Used in PCR

Primer Order	Sequence
**509 Forward**	5’- CAGTAAATGTATGGGGTCGCAG -3’
**509 Reverse**	5’- GGTGTCAGTGGGAGGAGGG -3’
**915 Forward**	5'- GTTATTTCCGTGGGATACTGAGAC-3'
**915 Reverse**	5'- GACCTCCTTGGCGTAGTAGTCG -3'

**Table 2.** Genotypes and Lengths of the Restriction Fragments After Enzymatic Digestion

Enzyme	Restriction Site	Genotype	Fragment Sizes
**Eco81I**	5'...C C▼T N A G G...3' 3'...G G A N T▲C C...5'	CC	36, 117
		CT	36, 117, 153
		TT	153
**BglI**	5'…GCCN NNN▼NGGC…3' 3'…CGGN▲NNN NCCG…5'	GG	212, 252, 60
		GC	212, 252, 312
		CC	212, 312

### 3.3. Statistical Analysis

Hardy-Weinberg equilibrium, allele distribution, genotype and frequency were analyzed by chi-square test. One sample Kolmogorov-Smirnov and Mann-Whitney tests were used to compare the cumulative distributions of the two data and the differences between two groups, respectively. Logistic regression was used for determination of OR/CI and adjustment of data for age and gender as confounder variables. All P values < 0.05 were considered significant. All statistical analyses were conducted using SPSS version 20 (SPSS Inc., Chicago, Illinois, The USA).

## 4. Results

**Figure 1.**
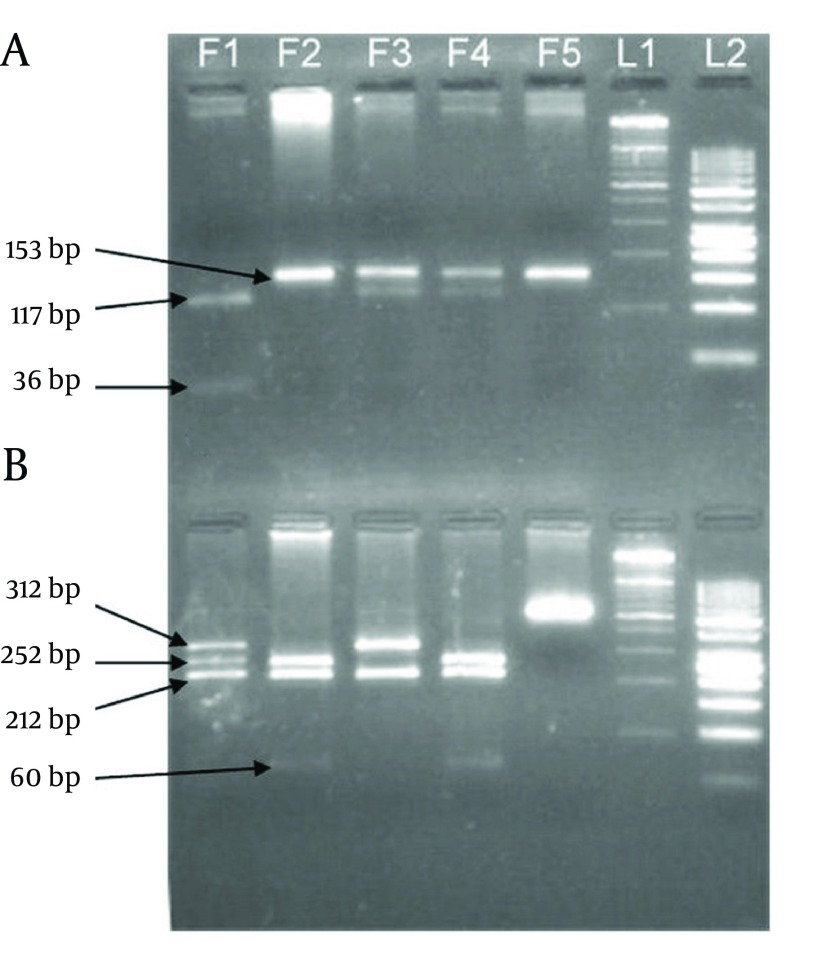
Products of Enzymatic Digestion and Their Fragments Size on Agarose Gel Electrophoresis A) Eco81I fragments (-509C/T polymorphism), B) BglI fragments (+915G/C polymorphism), F1) CC genotype of -509C/T and GC genotype of +915G/C, F2) TT genotype of -509C/T and GG genotype of +915G/C, F3) CT genotype of -509C/T and CC genotype of +915G/C, F4) Positive control, F5) Negative control (PCR product), L1) 100 bp DNA ladder, L2) 50 bp DNA ladder.

**Figure 2.**
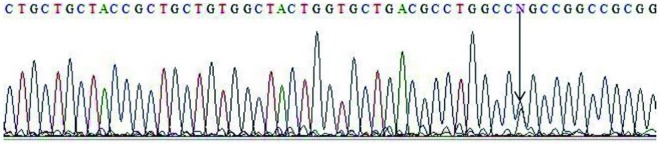
Sequence of Heterozygous +915T/C Arrow shows G and C alleles in this polymorphism

Electrophoresis patterns of PCR products and RFLP fragments are illustrated by Figure 1. The age ranges of case and control groups were 11 to 88 and 14 to 83, respectively. The mean age of the case group was 46.62 ± 17.105, and 43.38 ± 15.399 for the control group. Although among the 220 patients of the case group, male was the dominant gender (148 subjects), female gender was dominant in the healthy control group (128 subjects). The demographic data of two study groups are presented in Table 3. Logistic regression analyses were conducted to remove the confounding effects of age and gender. Genotype distribution and allele frequency of TGF-β1 -509C/T and +915T/C polymorphisms are shown in Table 4, whereas Table 5 depicts data separated for each gender. The frequency of both polymorphisms in the control group was in agreement with the Hardy-Weinberg equilibrium. The frequencies of the -509C/T genotypes CC, CT and TT were 22.7%, 52.7% and 24.5%, respectively, for the cases, and 29.5%, 44.1% and 26.4% respectively, for controls (P = 1.00, P = 0.101 and P = 0.318, respectively). The +915G/C genotype frequencies of GG, GC and CC were 87.7%, 10.5% and 1.8% respectively, for the cases, and 89.5%, 9.5% and 0.9%, respectively, for controls (P = 1.00, P = 0.266 and P = 0.472, respectively). The frequencies of -509C/T alleles C and T were 49.1% and 50.9%, respectively, for the cases and 51.6% and 48.4%, respectively, for controls (P = 1.00 and P = 0.304, respectively). The +915G/C allele frequencies of C and G were 93% and 7% respectively, for cases and 94.3% and 5.7%, respectively, for controls (P = 1.00 and P = 0.186 respectively). The results show that there is no significant difference among case and control groups, relating to these polymorphisms. Direct sequencing of PCR products confirmed the RFLP genotyping results. Sequencing electropherograms of PCR products with heterozygous genotype of +915T/C polymorphisms are shown in Figure 2. 

**Table 3.** The Demographic Data

	Case	Control
**Male, No.**	148	92
**Female, No.**	72	128
**Age Range, y**	11 to 88	14 to 83
**Age, Mean ± SD, y**	46.62 ± 17.105	43.38 ± 15.399

**Table 4.** Genotype Distribution and Allele Frequency of Two Studied TGF-β1 Polymorphisms ^a^, ^b^

Variable	Cases, (n = 194), No. (%)	Controls, (n = 246), No. (%)	Adjusted ^a^ OR (95% CI), P value
**-509C/T (rs1800469)**			
Genotypes			
CC	50 (22.7)	65 (29.5)	1.00
CT	116 (52.7)	97 (44.1)	0.674 (0.420-1.080), 0.101
TT	54 (24.5)	58 (26.4)	0.759 (0.442-1.304), 0.318
Alleles			
C	216 (49.1)	227 (51.6)	1.00
T	224 (50.9)	213 (48.4)	0.866 (0.659-1.139), 0.304
**+915G**/**C (rs1800471)**			
Genotypes			
GG	193 (87.7)	197 (89.5)	1.00
GC	23 (10.5)	21 (9.5)	0.368 (0.063-2.140), 0.266
CC	4 (1.8)	2 (0.9)	0.789 (0.413-1.507), 0.472
Alleles			
G	409 (93)	415 (94.3)	1.00
C	31 (7)	25 (5.7)	0.683 (0.388-1.201), 0.186

^a^ Adjusted for age and gender

^b^ OR, odds ratio; 95% CI, 95% confidence interval.

**Table 5.** Genotype Distribution and Allele Frequency of TGF-β1 Polymorphisms in Male and Female ^a^, ^b^

Variable	Male			Female		
	Cases, No. (%)	Controls, No. (%)	Adjusted ^a^ OR (95% CI), P value	Cases, No. (%)	Controls, No. (%)	Adjusted ^a^ OR (95% CI), P value
-509C/T, (rs1800469)						
Genotypes, No. (%)						
CC	32, (21.6)	29, (30.9)	1.00 (Reference)	18, (25)	36, (28.6)	1.00
CT	84, (56.8)	45, (47.9)	0.586 (0.315-1.090), 0.092	32, (44.4)	52, (41.3)	0.823 (0.400-1.694), 0.597
TT	32, (21.6)	20, (21.3)	0.680 (0.320-1.445), 0.316	22, (30.6)	38, (30.2)	0.867 (0.401-1.878), 0.718
Alleles, No. (%)						
C	148, (50)	103, (54.8)	1.00	68, (47.2)	124, (49.2)	1.00
T	148, (50)	85, (45.2)	0.820 (0.568-1.184),			
**+915G**/**C, (rs1800471)**						
Genotypes, No. (%)						
GG	133, (89.9)	87, (92.6)	1.00 (Reference)	60, (83.3)	110, (87.3)	1.00
GC	14, (9.5)	6, (6.4%)	1.569 (0.097-25.476), 0.751	9, (12.5)	15, (11.9)	0.167 (0.017-1.671), 0.128
CC	1, (0.7)	1, (1.1)	0.662 (0.245-1.792), 0.417	3, (4.2)	1, (0.8)	0.905 (0.374-2.193), 0.825
Alleles, No. (%)						
G	280, (94.6)	180, (95.7)	1.00 (Reference)	129, (89.6)	235, (93.3)	1.00
C	16, (5.4)	8, (4.3)	0.788 (0.330-1.882), 0.592	15, (10.4)	17, (6.7)	0.611 (0.295-1.268), 0.186

^a^ Adjusted for Age

^b^ OR, odds ratio; 95% CI, 95% confidence interval.

## 5. Discussion

The TGF-β1 plays several key roles during HBV infection. For example, it has a negative effect on HBV replication by decreasing HNF4A level, and also has indirect effects on immune responses to HBV infection. The HNF4A can increase pregenomic RNA (pgRNA) transcription, by loading on HNF4A binding elements (HNF4BEs) within HBV core promoter. Therefore, the increase in HNF4A enhances HBV replication. The expression of HNF4A is diminished in the presence of TGF-β1. Therefore, viral nucleocapsid formation and HBV replication are repressed after decreasing pgRNA (10). The TGF-β1 also acts as an important factor in peripheral regulatory T cells (Treg) development (23). Elevation of serum TGF-β1 level in a patient with chronic HBV can cause a significant increase in Treg frequency (24). It has been established that overactive immune responses can be restrained by Treg during chronic HBV infection, and therefore host tissue damage may be inhibited (25). As a result, TGF-β1 can play a crucial role in the course of chronic HBV infection.

The association of T allele of the -509C/T polymorphism with higher plasma levels of TGF-β1 has been demonstrated and luciferase assay studies have also shown higher transcriptional activity of this allele (13, 26). The +915G/C polymorphisms are associated with decreased plasma TGF-β1 level, as well. In the presence of the C allele, the production of TGF-β1 is lower than for the G allele (14, 16).

Recently, it has been revealed that serum TGF-β1 level is increased in Iranian patients with chronic HBV infection and it is not related to HBV viral copy load (27).

In 2003, Suzuki et al. showed that there is no genetic difference between Japanese healthy controls and patients with chronic hepatitis C with regard to +915G/C polymorphism (22).

In 2008, Pereira et al. analyzed eight polymorphisms in five different cytokine genes and just one of them (+915G/C polymorphism) was associated with chronic hepatitis C in Brazilian population (20).

Recently, Romani et al. (2011) analyzed a possible link between +915G/C, -509C/T, +869T/C polymorphisms and chronic HCV infection in Iranian population. They could not find any association between studied factors (21).

Liberek et al. (2009) proposed that TGF-β1 polymorphisms might not affect natural history and course of chronic hepatitis B and C diseases. However, the population size of this study was relatively small (19).

The present study showed there is no association between TGF-β1-509C/T and +915T/C polymorphisms and chronic hepatitis B in Iranian patients. The frequency of the GC and CC genotypes of the +915T/C polymorphism and the CT genotype of the -509C/T polymorphism in patient group was higher than in the control group. These results are in accordance with Falleti's survey (2008) on TGF-β1 genotypes association with cirrhosis in patients with chronic hepatitis B, the only exception being the CC genotype of the -509C/T polymorphism (28). Although the sample size of our study was slightly lower than Falleti's study, the results were similar. Therefore, it can be assumed that there is no relationship between this polymorphism and natural history/clinical course of chronic hepatitis B in the Iranian population (18). The main limitation of this study was that no measurement of serum TGF- β1 levels was performed, and also C allele of the +915T/C polymorphism is rare. Consequently, a study with a larger population size could possibly have a different result. Further, extended studies will be necessary for directly comparing the plasma TGF-β1 level of Iranian population with different genotypes of these polymorphisms.
