# Corrigendum to: Lack of Association Between Transforming Growth Factor Beta 1-509C/T and +915G/C Polymorphisms and Chronic Hepatitis B in Iranian Patients [Published in Hepatitis Monthly. 2014 May; 14 (5): e13100]

**DOI:** 10.5812/hepatmon.20548

**Published:** 2014-08-01

This corrigendum presents correct sentences of background and conclusions parts of abstract which contained two typographical errors.

Background: Chronic hepatitis B is one of the world's major health concerns.

Conclusions: There was no association between TGF-β1 -509C/T and +915G/C polymorphisms with chronic hepatitis B and it seems that these changes do not play a significant role in increasing the risk of chronic infection in Iranian patients.

Corresponding author:

Dr. Seyed Reza Mohebbi

